# Clinical Applications of Blood-Derived Extracellular Vesicle Biomarkers in Breast Cancer: A Scoping Review

**DOI:** 10.3390/ijms27104649

**Published:** 2026-05-21

**Authors:** Eun-Gyeong Lee, Kyung-Hee Kim, Se Bin Kim, Young Chan Chae, Min-Chae Kang, Sun-Young Kong

**Affiliations:** 1 Center for Breast Cancer, National Cancer Center, Goyang 10408, Republic of Korea; bnf333@ncc.re.kr; 2 Department of Cancer Biomedical Science, Graduate School of Cancer Science and Policy, National Cancer Center, Goyang 10408, Republic of Korea; 97209@ncc.re.kr; 3 Biopharmaceutical Chemistry Major, School of Applied Chemistry, Kookmin University, Seoul 02707, Republic of Korea; kyungheekim@kookmin.ac.kr; 4 Targeted Therapy Branch, National Cancer Center, Goyang 10408, Republic of Korea; 76864@ncc.re.kr; 5 Department of Biological Sciences, Ulsan National Institute of Science and Technology, Ulsan 44919, Republic of Korea; ychae@unist.ac.kr; 6 Department of Laboratory Medicine, National Cancer Center, Goyang 10408, Republic of Korea

**Keywords:** breast cancer, extracellular vesicles, exosomes, liquid biopsy, biomarkers, treatment response, prognosis, triple-negative breast cancer

## Abstract

Blood-derived extracellular vesicle (EV) biomarkers have emerged as promising liquid-biopsy analytes for monitoring treatment response and prognosis in breast cancer. This scoping review mapped the clinical evidence on blood-derived EV in breast cancer and identified key barriers to clinical translation. Following the Joanna Briggs Institute framework and PRISMA-ScR guidelines, we searched PubMed, Embase, and Web of Science for eligible studies published through November 2025. After duplicate removal, title and abstract screening, and full-text assessment, 64 clinical studies were included. Research activity increased markedly from 2020 onward, accounting for 87.5% (56/64) of included studies. The literature was concentrated in East Asia, particularly China (51.6%, 33/64). RNA-based biomarkers predominated (60.9%, 39/64), especially microRNAs (39.1%, 25/64). Prognostic outcomes were evaluated in 89.1% (57/64) of studies, treatment response in 51.6% (33/64), and both endpoints in 40.6% (26/64). Triple-negative breast cancer was the most frequently studied subtype in isolation (15.6%, 10/64). Methodological heterogeneity was substantial, and kit-based precipitation was the most common EV isolation method (57.8%, 37/64). EV biomarkers show promise for non-invasive monitoring in breast cancer, but methodological standardization, compliance with Minimal Information for Studies of Extracellular Vesicles guidelines, and large prospective validation studies remain necessary before routine clinical implementation.

## 1. Introduction

Breast cancer is the most frequently diagnosed malignancy and the leading cause of cancer-related death among women worldwide, with an estimated 2.3 million new cases and approximately 670,000 deaths in 2022 [1,2]. Breast cancer is a biologically heterogeneous disease comprising multiple molecular subtypes defined by distinct receptor expression profiles, including hormone receptor–positive (HR+), human epidermal growth factor receptor 2–positive (HER2+), and triple-negative breast cancer (TNBC). This molecular heterogeneity contributes to substantial variability in clinical behavior, treatment response, and long-term outcomes across patient populations. Despite major therapeutic advances, including targeted therapies, immune checkpoint inhibitors, and antibody–drug conjugates, treatment of aggressive subtypes such as TNBC and HER2+ breast cancer remains challenging because of tumor heterogeneity, acquired resistance, and limited predictive biomarkers [3]. Accurate, minimally invasive biomarkers that reflect real-time tumor biology throughout treatment are therefore needed.

Conventional serum tumor markers, including carcinoembryonic antigen (CEA) and cancer antigen 15-3 (CA15-3), have long been used in breast cancer management for disease monitoring and follow-up [4]. However, their clinical utility is limited by suboptimal sensitivity and specificity, particularly in early-stage disease and during longitudinal treatment monitoring [5]. These protein-based markers largely reflect tumor burden rather than molecular characteristics of the tumor and provide limited insight into tumor heterogeneity, treatment-induced biological changes, or microenvironmental dynamics [4,5]. Although tissue biopsy is informative, it is invasive, susceptible to sampling bias due to intratumoral heterogeneity, and impractical for serial monitoring. There is therefore an unmet need for biomarkers that can non-invasively capture the evolving molecular landscape of breast cancer in real time.

Liquid biopsy has emerged as a promising non-invasive strategy for real-time tumor surveillance and treatment monitoring [6]. Among liquid-biopsy analytes, circulating tumor DNA (ctDNA) and circulating tumor cells (CTCs) have been the most extensively studied [7]. However, both analytes have important limitations: they are often present at low abundance in early-stage disease, are vulnerable to rapid degradation, and provide limited insight into tumor–microenvironment interactions [7,8]. These limitations have increased interest in alternative liquid-biopsy analytes that may provide more comprehensive molecular information.

Extracellular vesicles (EVs), a heterogeneous population of membrane-enclosed nanoparticles that includes exosomes (30–150 nm), microvesicles (100–1000 nm), and apoptotic bodies, have emerged as promising sources of biomarkers in oncology [9]. EVs are actively secreted by viable tumor cells and released into the bloodstream, where their lipid bilayer membrane confers greater physicochemical stability than that of cell-free nucleic acids. Importantly, EVs carry diverse molecular cargo, including proteins, messenger RNA (mRNA), microRNA (miRNA), long non-coding RNA (lncRNA), DNA fragments, lipids, and glycans, that reflects the functional and transcriptional state of their cells of origin [10,11]. EVs are actively released by viable tumor and stromal cells and participate in intercellular communication through cargo transfer, thereby providing biologically relevant information regarding dynamic tumor behavior and tumor–microenvironment interactions. Unlike conventional serum tumor markers, which largely reflect tumor burden, EV-derived cargo may provide mechanistic insight into tumor heterogeneity, intercellular signaling, therapeutic resistance, and tumor–microenvironment interactions [9,12]. These properties make EVs attractive candidates for non-invasive, real-time monitoring of treatment response and prognostic stratification in breast cancer.

Despite the rapidly expanding literature on blood-derived EV biomarkers in breast cancer, the field remains highly heterogeneous with respect to EV isolation methods, biomarker types, analytical platforms, study populations, and clinical endpoints [10]. Furthermore, the extent to which EV-based biomarkers have been evaluated for clinical use, particularly for treatment response assessment and prognostic stratification across molecular subtypes, has not been comprehensively characterized. A systematic mapping of the available evidence is therefore needed to define the scope of existing research, identify areas of convergence and divergence, and delineate priorities for future translational studies.

This scoping review aimed to map the current evidence on blood-derived exosome and EV-based biomarkers in breast cancer. Specifically, we characterized the types of EV-derived biomarkers investigated, the isolation and analytical methods used, the clinical endpoints addressed, particularly those related to treatment-response monitoring and prognostic stratification, and the distribution of evidence across breast cancer subtypes. By providing a comprehensive evidence map, this review highlights current research gaps, informs future biomarker validation frameworks, and supports the development of a translational roadmap for EV-based liquid biopsy in precision breast cancer care.

## 2. Methods

### 2.1. Research Questions

This scoping review examined the evidence on blood-derived EV biomarkers for treatment response and prognosis in breast cancer. The review followed the methodological framework proposed by Arksey and O’Malley [13] and later refined by the Joanna Briggs Institute (JBI) [14]. To ensure transparent and comprehensive reporting, the review adhered to the Preferred Reporting Items for Systematic Reviews and Meta-Analyses extension for Scoping Reviews (PRISMA-ScR) guidelines [15]. The objectives of this review were to identify major research trends, summarize the clinical evidence across breast cancer subtypes, and delineate barriers to the clinical translation of EV-based biomarkers.

The following research questions guided this review: (1) What types of blood-derived EV biomarkers have been investigated in clinical studies of breast cancer, and what EV isolation and characterization methods were used? (2) To what extent have EV-derived biomarkers been evaluated for predicting treatment response, including pathological complete response and radiologic response, across breast cancer molecular subtypes? (3) Which EV biomarkers have been associated with prognostic outcomes, including overall survival, disease-free survival, and progression-free survival? (4) How is the current evidence distributed across breast cancer molecular subtypes, disease stages, and geographic regions, and what barriers remain to the clinical translation of EV-based liquid biopsy?

### 2.2. Eligibility Criteria

Study eligibility was defined according to the Population–Concept–Context (PCC) framework. The eligibility criteria are summarized in Table 1.

#### 2.2.1. Population

Eligible studies included human participants with breast cancer of any molecular subtype (e.g., HR-positive, HER2-positive, or TNBC) and at any disease stage, including early-stage, locally advanced, or metastatic disease.

#### 2.2.2. Concept

Eligible studies investigated biomarkers derived from blood-based EVs, including exosomes, microvesicles, and other EV subtypes, isolated from serum or plasma samples, regardless of size or biogenesis classification.

#### 2.2.3. Context

Eligible studies evaluated associations between EV-associated molecular cargo (e.g., proteins, miRNAs, and lncRNAs) and therapeutic or prognostic outcomes. Eligible endpoints included treatment response (e.g., pathological complete response [pCR] and radiologic response) and survival outcomes (e.g., overall survival [OS], disease-free survival [DFS], progression-free survival [PFS], and relapse-free survival [RFS]). All primary study designs, including prospective, retrospective, and interventional studies, were eligible. No date restrictions were applied.

Studies were excluded if they: (1) were conducted exclusively in cell lines or animal models without corresponding human clinical data; (2) focused solely on diagnostic or early detection performance without reporting therapeutic or prognostic outcomes; (3) included non-breast cancer populations; or (4) were published in languages other than English.

### 2.3. Search Strategy

A comprehensive literature search was conducted in three electronic databases, PubMed, Embase, and Web of Science, from database inception to 11 November 2025. The search strategy combined controlled vocabulary terms (MeSH for PubMed and Emtree for Embase) with free-text keywords across five core concepts: breast cancer, extracellular vesicles, biomarkers, clinical endpoints (treatment response, prognosis, survival, and recurrence), and sample source (blood, serum, and plasma). Wildcard operators (e.g., exosome* and biomarker*) were used in Web of Science to maximize search sensitivity. Publication-type filters were applied to exclude reviews, editorials, commentaries, and case reports. The complete search strategies for all three databases are provided in Supplementary Table S1. Grey literature was not systematically searched because the primary objective of this review was to synthesize peer-reviewed clinical evidence relevant to translational applicability.

The initial search yielded 1855 records (PubMed, 597; Embase, 295; Web of Science: 963). After duplicate removal, 1529 records remained for title and abstract screening, which was conducted independently by two reviewers (E.-G.L. and K.-H.K.). Full-text eligibility assessment was subsequently performed for 189 articles. Inter-rater agreement was assessed using Cohen’s kappa coefficient (κ = 0.778) for full-text inclusion decisions, indicating substantial agreement between the two reviewers. Discrepancies (*n* = 20, 10.6% of 189 full-text articles) were resolved through consensus discussion with adjudication by the corresponding author (S.-Y.K.), resulting in the final inclusion of 64 studies.

### 2.4. Study Selection and Data Charting

Study selection was conducted in three sequential stages: (1) identification, (2) screening, and (3) eligibility assessment. Following the database search, all identified records were imported into a reference-management tool for duplicate removal. Two independent reviewers screened the titles and abstracts of the remaining records according to the predefined eligibility criteria. For records that met the initial screening criteria, full-text articles were retrieved and independently reviewed for final inclusion. Articles excluded at this stage, together with the reasons for exclusion, are listed in Supplementary Table S2. Any disagreements between reviewers during study selection were resolved through consensus or consultation with a third reviewer (S.-Y.K.).

A standardized data-charting form was developed to extract relevant information from the included studies systematically. The extraction process captured key variables, including study characteristics (author, year, and design), clinical context (cohort size, breast cancer subtype, and treatment phase), EV methodology (sample source, isolation method, and characterization method), and clinical endpoints related to treatment response and prognosis.

Data extraction was performed independently by two reviewers (E.-G.L. and K.-H.K.), and discrepancies were resolved through discussion or adjudication by a third reviewer (S.-Y.K.).

For descriptive synthesis, each study was assigned to the RNA subtype representing its primary analytical focus.

### 2.5. Collating, Summarizing, and Reporting the Results

Consistent with the JBI methodological framework for scoping reviews, the charted data were collated and summarized using descriptive narrative synthesis. No meta-analysis or quantitative data pooling was performed because heterogeneity in study designs, EV isolation methods, biomarker types, and clinical endpoints precluded statistical synthesis.

The extracted data were organized into five thematic domains aligned with the review objectives: (1) publication trends and geographic distribution; (2) EV isolation and characterization methods, including compliance with the Minimal Information for Studies of Extracellular Vesicles (MISEV) guidelines; (3) distribution of breast cancer subtypes and disease stages; (4) composition and diversity of EV-derived biomarker types (e.g., miRNA, lncRNA, mRNA, circRNA, tRNA-derived fragments, proteins, lipids, DNA, and glycans); and (5) clinical endpoints, including treatment response (e.g., pCR and radiologic response) and prognostic outcomes (e.g., OS, DFS, RFS, and PFS). Findings were reported as counts and proportions, where applicable.

## 3. Results

### 3.1. Selection of Evidence

**Figure 1:**
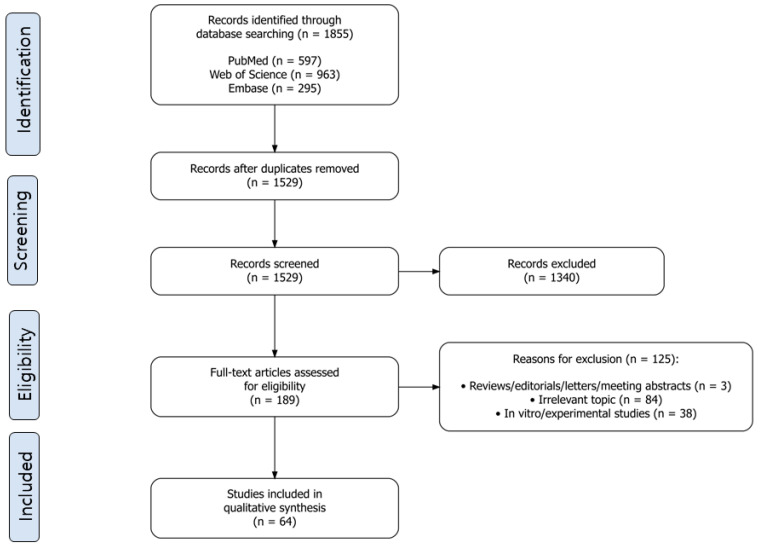
PRISMA flow diagram of the study search and selection process.

A total of 1855 records were initially identified through database searching. After duplicate removal, 1529 records remained for title and abstract screening by two independent reviewers. Based on the predefined eligibility criteria, 189 articles were retrieved for full-text assessment. Of these, 125 studies were excluded, and 64 studies were included in the final synthesis (Figure 1). Table 2 summarizes the key characteristics of the included studies, including breast cancer subtypes, EV isolation and characterization methods, molecular cargo types, and clinical endpoints. The main reasons for exclusion were absence of therapeutic or prognostic outcomes, use of non-blood biological samples (e.g., urine or breast milk), preclinical studies without corresponding human data, studies limited to diagnostic performance, inclusion of non–breast cancer populations, and conference abstracts without full-text reports.

### 3.2. Characteristics of Included Studies

Table 3 summarizes the major characteristics of the included studies according to EV biomarker type, molecular subtype, and clinical application.

#### 3.2.1. Publication Year and Geographic Distribution

**Figure 2:**
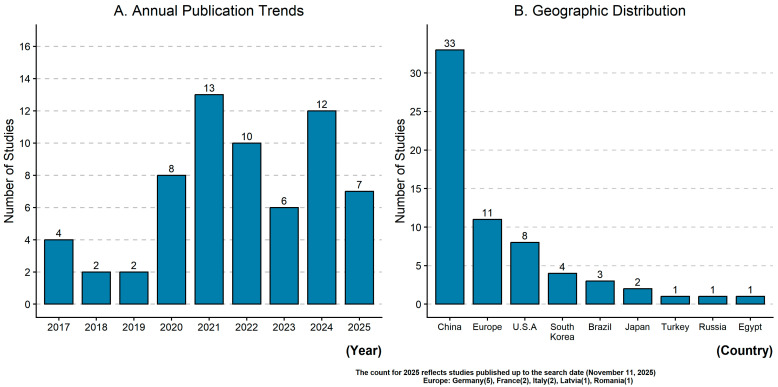
Temporal and geographic distribution of the included studies (*n* = 64). (**A**) Annual number of publications on blood-derived extracellular vesicle biomarkers in breast cancer from 2017 to 2025. (**B**) Geographic distribution of the included studies according to the country or region of the primary study cohort.

Most of the included studies were published after 2020 (87.5%, 56/64) [16,17,18,19,20,21,22,23,24,25,26,27,28,29,30,31,32,33,34,35,36,37,38,39,40,41,42,43,44,45,46,47,48,49,50,51,52,53,54,55,56,57,58,59,60,61,62,63,64,65,66,67,68,69,70,71]. The remaining eight studies (12.5%) were published between 2017 and 2019 [72,73,74,75,76,77,78,79]. Geographically, most studies were conducted in East Asia, particularly China (*n* = 33) [18,22,25,34,35,36,37,38,39,40,41,42,43,44,45,48,49,50,52,55,61,62,63,65,66,67,68,69,70,71,74,75,79,80], followed by Europe (*n* = 11) [17,23,26,28,46,47,56,72,76,77,78] and the United States (*n* = 8) [16,21,24,29,57,59,60,64]. Additional studies were conducted in South Korea (*n* = 4) [30,31,32,33], Brazil (*n* = 3) [19,20,54], Japan (*n* = 2) [51,73], Turkey (*n* = 1) [27], Russia (*n* = 1) [58], and Egypt (*n* = 1) [53] (Figure 2).

**Table 2:** Summary of included studies (*n* = 64).

No.	Author (Year)	Biomarker Type	Clinical Endpoint	Molecular Subtype/Target Population	Key Biomarker(s)	Main Clinical Association	Sample (Method)	N
1	Curtaz (2022) [23]	**miRNA**	**Prognosis**	All subtypes	hsa-miR-576-3p, miR-130a-3p	Brain metastasis, grading	Serum (Kit)	65
2	Liu (2020) [43]	**mRNA**	**Both**	TNBC-focused	FBXO39 mRNA	Monitoring, OS, lymph node metastasis	Serum (UC + Kit)	100
3	Alvarez (2022) [16]	**Protein**	**Response**	All subtypes	5-protein panel (GPIBA, etc.)	pCR	Plasma (SEC/PPLC)	17
4	Yuan (2021) [69]	**miRNA**	**Prognosis**	HER2+ only	hsa-miR-21, PDCD4	Bone metastasis	Serum (Kit)	51
5	Li (2024) [38]	**miRNA**	**Both**	TNBC only	cirmiR-20a-5p, NPAT	Anti-PD-1 sensitivity, survival	Plasma (UC)	50
6	Shen (2021) [48]	**miRNA**	**Prognosis**	All subtypes	miR-7641	OS, DFS	Plasma (UC)	28
7	Fan (2025) [25]	**Protein**	**Prognosis**	All subtypes	ITGB2	OS, DFS	Serum (Kit)	212
8	Todorova (2022) [57]	**miRNA**	**Response**	All subtypes	miR-30b, miR-328, miR-423, miR-127	pCR, RFS	Plasma (Kit)	20
9	Wang (2021) [61]	**miRNA**	**Prognosis**	HR+/HER2− disease	miR-363-5p, PDGFB	PFS	Plasma (UC)	10
10	Tkach (2022) [56]	**Protein**	**Response**	HR+ only	CD326, CD146, CD105	Clinical response	Plasma (SEC)	27
11	Baldasici (2022) [17]	**lncRNA**	**Response**	All subtypes	HOTAIR, MALAT1	pCR	Plasma (Kit)	72
12	Desai (2022) [24]	**Protein**	**Response**	TNBC only	Annexin A2 (AnxA2)	Treatment response	Serum (Kit)	17
13	Sadovska (2022) [47]	**miRNA**	**Prognosis**	TNBC-focused	miR-155, miR-181a, miR-181b	RFS, OS	Plasma (SEC)	32
14	Li (2021) [36]	**miRNA**	**Response**	All subtypes	miR-3662, miR-146a, miR-1290	Treatment response	Serum (Kit)	60
15	Fontana (2025) [26]	**miRNA**	**Prognosis**	All subtypes	miR-3916, miR-3162-3p	OS, RFS	Plasma (Kit)	296
16	Cui (2020) [22]	**mRNA**	**Prognosis**	All subtypes	LDHC mRNA	OS, recurrence	Serum (Kit)	75
17	Ni (2018) [76]	**miRNA**	**Prognosis**	All subtypes	miR-16, miR-93, miR-494	OS, recurrence	Plasma (Kit)	153
18	Sueta (2017) [73]	**miRNA**	**Prognosis**	All subtypes	miR-340, miR-17, miR-130a	OS, DFS	Serum (Kit)	32
19	Wu (2020) [63]	**miRNA**	**Prognosis**	All subtypes	miR-150-5p, miR-576-3p	OS, recurrence	Plasma (Kit)	27
20	Tamarindo (2025) [54]	**Protein**	**Prognosis**	TNBC only	HISTH2A, CSTA, HISTH2B	OS	Plasma (SEC)	29
21	Kim (2024) [32]	**miRNA, Protein**	**Both**	All subtypes	MDR1, miR-21, miR-221	pCR, PFS	Plasma (UC)	36
22	Jung (2021) [31]	**Protein**	**Response**	All subtypes	NGF, IP-10, MMP-1	Clinical response	Serum (UC)	129
23	König (2017) [72]	**DNA**	**Both**	All subtypes	EV-associated cfDNA	Clinical response, OS	Plasma (Kit)	105
24	Causin (2024) [20]	**miRNA**	**Prognosis**	All subtypes	miR-19a-3p, miR-130b-3p	OS	Plasma (UC)	24
25	Li (2021) [37]	**Protein**	**Both**	TNBC only	Annexin A6	Clinical response	Serum (Kit)	21
26	Zhuang (2024) [71]	**circRNA**	**Prognosis**	All subtypes	circ-0100519	OS, DFS	Serum (UC)	20
27	Liu (2023) [41]	**lncRNA**	**Both**	HER2+ only	Linc00969	Response monitoring	Serum/Plasma (UC)	108
28	Wu (2021) [64]	**miRNA, Protein**	**Prognosis**	HR+/TNBC	miR-19a, IBSP	OS, bone metastasis	Serum (UC)	87
29	Li (2024) [39]	**miRNA**	**Prognosis**	All subtypes	miR-361-3p	OS, metastasis	Plasma (Kit)	37
30	Zhang (2020) [70]	**miRNA**	**Both**	HER2+ only	miR-1246, miR-155	Response, survival	Plasma (Kit)	183
31	Sueta (2021) [51]	**miRNA**	**Both**	TNBC only	miR-4448, miR-2392	pCR, OS	Serum (Kit)	24
32	Sun (2023) [52]	**tRF**	**Both**	HR+ only	tRF-16-K8J7K1B	Response, DFS	Serum (UC)	56
33	Kim (2024) [33]	**miRNA**	**Both**	All subtypes	5-miRNA signature	Response, OS, DFS	Plasma (Immunoaffinity)	35
34	Bao (2021) [18]	**miRNA**	**Prognosis**	All subtypes	miGISig (3-miRNA panel)	OS, DRFS	Serum/Plasma (UC)	>1000
35	Shi (2022) [49]	**lncRNA**	**Prognosis**	All subtypes	lncRNA DANCR	OS	Serum (Kit)	120
36	Li (2020) [35]	**miRNA**	**Prognosis**	N/A	miR-148a	OS	Serum (Kit)	125
37	Wang (2017) [74]	**Protein**	**Both**	All subtypes	TRPC5	Response	Plasma (Kit)	131
38	Niu (2025) [45]	**lncRNA, miRNA**	**Both**	TNBC/HER2+/Luminal	LINC00899, miR-425	Response	Plasma (Kit)	119
39	Carvalho (2022) [19]	**miRNA**	**Prognosis**	TNBC-focused	4-miRNA panel (miR-142, etc.)	OS	Serum (Kit)	150
40	Richard (2024) [46]	**Lipid**	**Both**	HR+ only	16 EV-sphingo scores	Response	Plasma (SEC)	44
41	Jiang (2024) [29]	**Protein**	**Both**	HER2+/TNBC	HER2-enriched EVs	Response	Plasma (SEC)	11
42	Tang (2019) [79]	**lncRNA**	**Both**	All subtypes	lncRNA HOTAIR	Response	Serum (Kit)	65
43	Del Re (2019) [78]	**mRNA**	**Both**	HR+ only	TK1, CDK9 mRNA	Response	Plasma (Kit)	40
44	Yang (2024) [67]	**miRNA**	**Prognosis**	All subtypes	miR-203a-3p	OS	Plasma (Kit)	45
45	Su (2021) [50]	**mRNA, miRNA**	**Both**	All subtypes	11-exLR signature	Response	Plasma (Kit)	112
46	Yang (2025) [68]	**Protein**	**Both**	HER2+ focus	Exosomal HER2	Response	Plasma (Kit)	51
47	Yang (2017) [75]	**Protein**	**Both**	All subtypes	GSTP1	Response	Serum (UC)	30
48	Vikramdeo (2023) [59]	**DNA**	**Prognosis**	TNBC only	EV-mtDNA mutations	OS, DFS	Plasma (Kit)	32
49	Eskiler (2023) [27]	**mRNA**	**Both**	All subtypes	FGFR2, FGFR3 mRNA	Response	Serum (Kit)	25
50	Hoffmann (2023) [28]	**Protein**	**Both**	TNBC only	PD-L2 EVs	Response	Plasma (UC)	54
51	Tian (2021) [55]	**Protein**	**Both**	All subtypes	8-EV protein signature	Response	Plasma (UC)	85
52	Vinik (2020) [60]	**Protein**	**Prognosis**	All subtypes	FAK, Fibronectin	OS, DFS	Plasma (SEC)	46
53	Tutanov (2020) [58]	**Protein**	**Prognosis**	All subtypes	SOCS3, IGF2R, FAK	OS	Plasma (UC)	23
54	Xu (2024) [66]	**Protein**	**Prognosis**	All subtypes	TALDO1	OS	Serum (UC)	126
55	Jung (2023) [30]	**Protein**	**Both**	TNBC only	APRIL, CXCL13, VEGF	pCR, DFS	Serum (Kit)	190
56	Talat (2025) [53]	**Protein**	**Prognosis**	All subtypes	SDC2, Fibronectin	OS	Plasma (UC)	169
57	Lan (2021) [34]	**lncRNA**	**Prognosis**	TNBC only	lncRNA XIST	OS	Serum (Kit)	91
58	Chaudhary (2020) [21]	**Protein**	**Prognosis**	All subtypes	Annexin A2	OS, DFS	Serum (Kit)	169
59	Stevic (2018) [77]	**miRNA**	**Both**	TNBC/HER2+	miR-27a, miR-30e, miR-155	pCR	Plasma (Kit)	435
60	Wang (2025) [80]	**Protein**	**Both**	TNBC only	RTN4	Response	Plasma (UC)	104
61	Li (2024) [40]	**Glycan**	**Both**	TNBC-focused	EV glycan signature	Response	Plasma (SEC)	72
62	Xu (2024) [65]	**Protein**	**Prognosis**	All subtypes	TEVs (CD63+/EpCAM+)	OS	Serum (Capture)	512
63	Na-er (2021) [44]	**lncRNA**	**Prognosis**	TNBC-focused	SUMO1P3	OS	Serum (Kit)	190
64	Liu (2022) [42]	**circRNA**	**Prognosis**	All subtypes	hsa_circ_0058514	OS	Plasma (Kit)	135

Abbreviations: N, number; BC, Breast cancer; HR+, hormone receptor–positive; HER2+, human epidermal growth factor receptor 2–positive; TNBC, triple-negative breast cancer; N/A, Not Applicable; mRNA, messenger RNA; miRNA, microRNA; lncRNA, long non-coding RNA; circRNA, circular RNA; tRF, transfer RNA–derived fragments; UC, Ultracentrifugation; SEC, Size-Exclusion Chromatography; pCR, pathological complete response; OS, overall survival; DFS, disease-free survival.

**Table 3:** Summary of Included Studies According to EV Biomarker Type and Clinical Application.

Category	Subcategory	No. of Studies (%)	References
**EV isolation method**	Kit-based/precipitation	36 (56.2%)	[17,19,21,22,23,24,25,26,27,28,30,34,35,36,37,39,42,44,45,49,50,51,57,59,63,67,68,69,70,72,73,74,76,77,78,79]
	Ultracentrifugation (UC)	18 (28.1%)	[18,20,31,32,38,41,43,48,52,53,55,58,61,64,66,71,75,80]
	Size-exclusion chromatography (SEC)	8 (12.5%)	[16,29,40,46,47,54,56,60]
	Other specialized methods	2 (3.1%)	[33,65]
Biological sample source *	Plasma	38 (59.4%)	[16,17,18,20,26,28,29,32,33,38,39,40,41,42,45,46,47,48,50,53,54,55,56,57,58,59,60,61,63,67,68,70,72,74,76,77,78,80]
	Serum	28 (43.8%)	[18,19,21,22,23,24,25,27,30,31,34,35,36,37,41,43,44,49,51,52,64,65,66,69,71,73,75,79]
**EV characterization methods**	Western blotting (WB)	48 (75.0%)	Supplementary Table S3
	Nanoparticle tracking analysis (NTA)	41 (64.1%)	Supplementary Table S3
	Transmission electron microscopy (TEM)	36 (56.2%)	Supplementary Table S3
**EV-associated markers**	CD63	41 (64.1%)	Supplementary Table S3
	CD9	27 (42.2%)	Supplementary Table S3
	TSG101	25 (39.1%)	Supplementary Table S3
	CD81	17 (26.6%)	Supplementary Table S3
Molecular subtype focus *	TNBC	10 (15.6%)	[24,28,30,34,37,38,51,54,59,80]
	HR+/luminal	5 (7.8%)	[46,52,56,61,78]
	HER2 +	2 (3.1%)	[41,70]
Disease stage *	Early-stage/ locally advanced	31 (48.4%)	[16,20,22,25,27,28,29,30,31,32,34,36,42,44,45,47,51,52,53,54,57,58,60,61,67,69,72,73,76,77,79]
	Stage IV	29 (45.3%)	[17,18,19,23,24,26,33,35,37,39,40,41,43,46,48,49,50,55,56,59,64,65,66,68,70,71,74,75,78]
	Mixed non-metastatic and metastatic	9 (14.1%)	[22,38,43,46,51,55,57,69,70]
**Biomarker category**	RNA-based biomarkers	39 (60.9%)	[17,18,19,20,22,23,26,27,32,33,34,35,36,38,39,41,42,43,44,45,47,48,49,50,51,52,57,61,63,64,67,69,70,71,73,76,77,78,79]
	miRNA	25 (39.1%)	[18,19,20,23,26,32,33,35,36,38,39,45,47,48,51,57,61,63,64,67,69,70,73,76,77]
	lncRNA	7 (10.9%)	[17,34,41,44,45,49,79]
	mRNA	4 (6.3%)	[22,27,43,78]
	circRNA	2 (3.1%)	[42,71]
	tRF	1 (1.6%)	[52]
	Protein-based biomarkers	21 (32.8%)	[16,21,24,25,28,29,30,31,37,53,54,55,56,58,60,65,66,68,74,75,80]
	DNA-based biomarkers	2 (3.1%)	[59,72]
	Lipid-based biomarkers	1 (1.6%)	[46]
	Glycan-based biomarkers	1 (1.6%)	[40]
Clinical endpoints *	Treatment response	33 (51.6%)	[16,17,24,27,28,29,30,31,32,33,36,37,38,40,41,43,45,46,50,52,55,56,57,68,70,72,74,75,77,78,79,80]
	Prognostic outcomes	57 (89.1%)	[18,19,20,21,22,23,25,26,27,28,29,30,32,33,34,35,37,38,39,40,41,42,43,44,45,46,47,48,49,50,51,52,53,54,55,58,59,60,61,63,64,65,66,67,68,69,70,71,72,73,74,75,76,77,78,79,80]
	Both response and prognosis	26 (40.6%)	[27,28,29,30,32,33,37,38,40,41,45,46,50,51,52,55,68,70,72,74,75,77,78,79,80]
**Treatment response**	pCR assessment	14 (21.9%)	[16,17,27,30,32,33,40,45,47,50,51,57,68,77]
	Longitudinal monitoring	6 (9.4%)	[22,43,46,51,55,57]

Abbreviations: HR+, hormone receptor–positive; HER2+, human epidermal growth factor receptor 2–positive; TNBC, triple-negative breast cancer; mRNA, messenger RNA; miRNA, microRNA; lncRNA, long non-coding RNA; tRF, transfer RNA–derived fragments; pCR, pathological complete response, * Percentages may exceed 100% because some studies evaluated multiple biomarker categories, biological sample sources, disease stages, molecular subtypes, or clinical endpoints.

#### 3.2.2. EV Isolation and Characterization Methods

Among EV isolation approaches, kit-based or precipitation methods were used most frequently (56.2%, 36/64) [17,19,21,22,23,24,25,26,27,28,30,34,35,36,37,39,42,44,45,49,50,51,57,59,63,67,68,69,70,72,73,74,76,77,78,79], followed by ultracentrifugation (UC) (28.1%, 18/64) [18,20,31,32,38,41,43,48,52,53,55,58,61,64,66,71,75,80]. The remaining studies used size-exclusion chromatography (12.5%, 8/64) [16,29,40,46,47,54,56,60] or other specialized platforms (3.1%, 2/64) [33,65], such as immunoaffinity capture.

For biological sample type, plasma was used in 59.4% (38/64) of studies [16,17,18,20,26,28,29,32,33,38,39,40,41,42,45,46,47,48,50,53,54,55,56,57,58,59,60,61,63,67,68,70,72,74,76,77,78,80], whereas serum was used in 43.8% (28/64) [18,19,21,22,23,24,25,27,30,31,34,35,36,37,41,43,44,49,51,52,64,65,66,69,71,73,75,79], including studies that used both sources.

Of the 64 included studies, 53 (82.8%) reported EV characterization consistent with the MISEV guidelines [81]. The most commonly reported methods were Western blotting (WB; 75.0%, 48/64), nanoparticle tracking analysis (NTA; 64.1%, 41/64), and transmission electron microscopy (TEM; 56.2%, 36/64). The most frequently reported EV-associated markers were CD63 (64.1%, 41/64), CD9 (42.2%, 27/64), TSG101 (39.1%, 25/64), and CD81 (26.6%, 17/64). The remaining 11 studies (17.2%) did not report EV-associated markers. Study-level isolation protocols and MISEV-related reporting details are summarized in Supplementary Table S3.

#### 3.2.3. Breast Cancer Subtype and Disease Stage

In addition to studies involving heterogeneous breast cancer populations, some studies focused on specific molecular subtypes. Seventeen studies (26.6%) focused exclusively on a single molecular subtype. Among these, TNBC was the most common exclusive subtype (15.6%, 10/64) [24,28,30,34,37,38,51,54,59,80], followed by HR-positive/luminal disease (7.8%, 5/64) [46,52,56,61,78] and HER2-positive disease (3.1%, 2/64) [41,70]. The remaining 47 studies (73.4%) included either all subtypes or mixed subtype populations.

Regarding disease stage, 48.4% (31/64) of studies included patients with early-stage or locally advanced breast cancer [16,20,22,25,27,28,29,30,31,32,34,36,42,44,45,47,51,52,53,54,57,58,60,61,67,69,72,73,76,77,79], whereas 45.3% (29/64) included patients with stage IV disease [17,18,19,23,24,26,33,35,37,39,40,41,43,46,48,49,50,55,56,59,64,65,66,68,70,71,74,75,78]. Nine studies (14.1%) [22,38,43,46,51,55,57,69,70] included patients across both non-metastatic and metastatic settings. Table 4 summarizes EV biomarkers reported across breast cancer subtypes. Subtype-specific studies reported biomarkers associated with distinct clinical contexts, including endocrine resistance in HR-positive disease and immune-related features in TNBC.

### 3.3. Biomarker Composition

#### 3.3.1. Distribution of EV Biomarkers

The biomarkers identified across the 64 included studies comprised a broad range of EV cargo molecules, with RNA-based biomarkers representing the largest category. Overall, 39 studies (60.9%) [17,18,19,20,22,23,26,27,32,33,34,35,36,38,39,41,42,43,44,45,47,48,49,50,51,52,57,61,63,64,67,69,70,71,73,76,77,78,79] investigated RNA molecules as the primary biomarker class. Within the RNA category, microRNAs (miRNAs) were the most frequently studied subtype, accounting for 39.1% (25/64) of all included studies [18,19,20,23,26,32,33,35,36,38,39,45,47,48,51,57,61,63,64,67,69,70,73,76,77]. Other RNA species included long non-coding RNAs (lncRNA; 10.9%, 7/64) [17,34,41,44,45,49,79], messenger RNAs (mRNA; 6.3%, 4/64) [22,27,43,78], circular RNAs (circRNA; 3.1%, 2/64) [42,71], and transfer RNA-derived fragments (tRF; 1.6%, 1/64) [52].

Protein-based biomarkers were evaluated in 32.8% (21/64) of included studies [16,21,24,25,28,29,30,31,37,53,54,55,56,58,60,65,66,68,74,75,80]. Some studies adopted a multi-analyte approach, profiling EV proteins together with RNA-based markers. For example, Kim et al. [32] and Wu et al. [64] analyzed both EV proteins and miRNAs within the same study. In some studies, protein-based signatures were combined with RNA cargo in multimarker panels. DNA-based biomarkers were evaluated in 3.1% (2/64) of included studies, including EV-associated cell-free DNA [72] and mitochondrial DNA mutations [59]. One study evaluated lipid-based EV cargo (1.6%, 1/64) [46] and another evaluated glycan-based EV cargo (1.6%, 1/64) [40].

#### 3.3.2. miRNA and Other RNA-Based Biomarkers

miRNAs were the most frequently studied EV cargo (39.1%, 25/64). Frequently reported miRNA biomarkers included miR-155, miR-21, and miR-1246, which were reported in association with treatment response and survival outcomes across multiple subtypes [32,64]. LncRNAs (10.9%, 7/64) [17,34,41,44,45,49,79] and circRNAs (3.1%, 2/64) [42,71] were also evaluated as biomarkers in studies of therapeutic resistance and disease progression. mRNA-based biomarkers were evaluated in four studies (6.3%), including FBXO39 mRNA, LDHC mRNA, TK1/CDK9 mRNA, and FGFR2/FGFR3 mRNA [22,27,43,78]. One study evaluated tRNA-derived fragments as EV-based biomarkers [52].

#### 3.3.3. Protein-Based and DNA-Based Biomarkers

Among the 21 studies (32.8%) evaluating EV proteins, both broad proteomic profiling and targeted surface-marker approaches were reported. Reported protein biomarkers included GSTP1 [75], Annexin A2 [24], and MDR1 [32]. DNA-based investigations (3.1%, 2/64) included analyses of mitochondrial DNA mutations in EVs [59] and EV-associated cell-free DNA (cfDNA) [72].

After characterizing the distribution and composition of EV-associated biomarkers, we next examined their reported clinical applications, including treatment response monitoring and prognostic stratification.

### 3.4. Clinical Outcomes

#### 3.4.1. Distribution of Clinical Endpoints

The clinical endpoints evaluated across the included studies were categorized as treatment response or prognosis. Based on the primary analytical focus, 33 studies (51.6%) [16,17,24,27,28,29,30,31,32,33,36,37,38,40,41,43,45,46,50,52,55,56,57,68,70,72,74,75,77,78,79,80] evaluated treatment response, whereas 57 studies (89.1%) [18,19,20,21,22,23,25,26,27,28,29,30,32,33,34,35,37,38,39,40,41,42,43,44,45,46,47,48,49,50,51,52,53,54,55,58,59,60,61,63,64,65,66,67,68,69,70,71,72,73,74,75,76,77,78,79,80] evaluated prognostic outcomes. Twenty-six studies (40.6%) [27,28,29,30,32,33,37,38,40,41,45,46,50,51,52,55,68,70,72,74,75,77,78,79,80] evaluated both treatment response and prognostic outcomes within the same study. Because of this overlap, the total number of endpoint categories exceeds the number of included studies.

#### 3.4.2. Treatment Response and Monitoring

Of the 33 studies evaluating treatment response, 14 (21.9%) [16,17,27,30,32,33,40,45,47,50,51,57,68,77] specifically assessed pathological complete response (pCR) after neoadjuvant chemotherapy. Longitudinal sampling was reported in six studies to monitor treatment response over time (Table 5). Several studies reported decreases in specific EV cargo levels after surgery or early cycles of chemotherapy in association with favorable outcomes. For example, Liu et al. [43] reported that postoperative decreases in EV-associated FBXO39 mRNA levels were associated with improved treatment response. Similarly, Todorova et al. [57] reported that changes in circulating miRNA profiles, including miR-141 and miR-182, after the first cycle of neoadjuvant chemotherapy were associated with pCR.

Some studies also evaluated EV-derived biomarkers for early detection of treatment resistance. Richard et al. [46] reported that alterations in EV-associated sphingolipid signatures after two months of CDK4/6 inhibitor therapy were associated with early drug resistance in hormone receptor–positive metastatic breast cancer.

#### 3.4.3. Prognostic Indicators and Survival Outcomes

Associations between EV biomarkers and prognostic outcomes were reported in 57 studies, including 31 prognosis-only studies and 26 studies that also evaluated treatment response. The most frequently analyzed prognostic endpoint was overall survival (OS; *n* = 32) [18,19,20,21,22,25,26,32,34,35,42,43,44,47,48,49,51,53,54,58,59,60,63,64,65,66,67,72,73,76], followed by DFS/RFS (*n* = 16) [18,21,22,25,26,30,47,48,52,59,60,71,73,76] and PFS (*n* = 2) [32,61]. Because some studies reported more than one survival endpoint, the total number of reported survival endpoints exceeds the number of prognosis-focused studies. Some studies reported associations in high-risk subgroups, particularly TNBC and HER2-positive disease. [32,47,70] Beyond survival endpoints, some EV signatures were reported in association with organ-specific metastatic risk, including brain [23] and bone metastases [69].

Reported OS-associated miRNA biomarkers included miR-7641 [48], miR-361-3p [39], miR-148a [35], and miR-203a-3p [67]. Multi-miRNA panels were also reported, including miGISig (miR-421, miR-128-3p, and miR-200c) [18] and a four-miRNA panel comprising miR-142-5p, miR-150-5p, miR-320a-3p, and miR-4433b-5p [19]. Beyond miRNAs, reported protein-based prognostic biomarkers included a seven-protein signature anchored by FAK and fibronectin [60], an 11-protein panel including SOCS3 and IGF2R [58], EV-associated TALDO1 [66], and Annexin A2 [21], each reported in association with OS or DFS. Non-coding RNA biomarkers beyond miRNAs included lncRNA DANCR [49], lncRNA XIST [34], lncRNA SUMO1P3 [44], and circRNA hsa_circ_0058514 [42], each reported in association with OS.

## 4. Discussion

This scoping review examined the available evidence on blood-derived EV biomarkers for treatment response and prognosis in breast cancer. Among the 64 included studies, 87.5% (56/64) were published from 2020 onwards, indicating increased recent research activity in this field.

RNA-based biomarkers were the largest category (60.9%, 39/64), with miRNAs representing the most frequently studied cargo type (39.1%, 25/64) across multiple breast cancer subtypes. Recent studies have also incorporated protein-based markers and multimodal approaches, suggesting increasing diversification of EV biomarker research. Treatment response was evaluated in 33 studies (51.6%), prognostic outcomes in 57 studies (89.1%), and both endpoints in 26 studies (40.6%).

### 4.1. Clinical Rationale for EV-Based Liquid Biopsy in Breast Cancer

Conventional serum tumor markers, including CEA and CA15-3, have long been used in breast cancer management for disease monitoring and follow-up. However, their clinical utility remains limited by suboptimal sensitivity and specificity, particularly in early-stage disease. In patients with stage I–III invasive breast cancer, elevated preoperative CEA and CA15-3 levels have been reported in only 10.9% and 13.9% of cases, respectively, supporting their limited sensitivity in early-stage disease detection [82]. In the metastatic setting, elevated CA15-3 and CEA levels at initial diagnosis of recurrence have been reported in approximately 57.4% and 34.2% of patients, respectively [83].

EV-based biomarkers have emerged as a promising liquid-biopsy analyte, offering potential advantages over conventional analytes such as ctDNA and CTCs. A key structural feature is the lipid bilayer membrane of EVs, which may enhance molecular stability by protecting encapsulated cargo from enzymatic degradation in the circulation. Unlike markers that primarily reflect tumor burden, EVs are released by viable tumor cells and may provide information on the dynamic functional state of the tumor ecosystem [7,62,84].

Potential advantages of EVs can be considered across four mechanistic dimensions. First, with respect to tumor heterogeneity, EV cargo may reflect the transcriptional and proteomic diversity of viable tumor cells. Unlike ctDNA, which primarily captures genomic alterations, EV-associated signals may change during treatment and may therefore provide complementary information of temporal heterogeneity relative to CTC profiles [85]. Second, EVs participate in intercellular signaling by transporting bioactive cargo that can alter signaling pathways in recipient cells. This bidirectional communication between tumor cells and the surrounding stroma, including macrophages, fibroblasts, and endothelial cells, may capture biological interactions not readily assessed by cell-free nucleic acid approaches alone [86]. Third, EVs may contribute to therapeutic resistance signaling; for example, intercellular delivery of EV-associated Annexin A6 or PKM2 has been implicated in drug resistance in neighboring cells [37]. Accordingly, detection of such cargo may provide earlier or more mechanistically informative signals of emerging therapeutic failure than conventional radiological endpoints. Finally, EVs have been implicated in remodeling the tumor microenvironment and in the formation of pre-metastatic niches in distant organs such as the lung, bone, and brain [87].

The multidimensional molecular cargo of EVs, spanning miRNAs, lncRNAs, proteins, and DNA, also supports the development of multimarker panels that may improve predictive and prognostic performance relative to single-analyte approaches. Several studies included in this review reported improved discriminatory performance when EV protein and RNA cargo were integrated rather than evaluated separately [24,32]. Collectively, these properties support the rationale for continued evaluation of EVs as informative biomarker sources in breast cancer.

### 4.2. Bridging the Technical Gap: Purity Versus Throughput

A key methodological challenge identified in this review is the trade-off between EV isolation purity and clinical scalability. According to Supplementary Table S3, kit-based precipitation methods were the most frequently used approach (57.8%, 37/64), likely because of their operational simplicity and compatibility with high-throughput processing. However, these methods are susceptible to co-isolation of non-vesicular protein contaminants, which may compromise analytical specificity and reproducibility.

Ultracentrifugation (UC) was used in 28.1% (18/64) of studies and is commonly regarded as the reference technique because it provides comparatively higher isolation purity. However, its requirement for specialized equipment and extended processing times limits its feasibility for routine clinical implementation. Size-exclusion chromatography (SEC) was used in 12.5% (8/64) of studies and may offer an intermediate balance between purity and scalability. The remaining studies (3.1%, 2/64) used specialized platforms such as immunoaffinity capture and direct vesicle-capture assays.

These methodological differences likely contribute to interstudy heterogeneity [88,89]. Emerging microfluidic platforms and nanosensor-based technologies may help address this gap by enabling rapid, high-sensitivity EV detection with minimal preprocessing, thereby improving the clinical feasibility of EV-based biomarker assays [90].

### 4.3. Standardization Challenges and MISEV Compliance

Methodological standardization remains a key prerequisite for clinical translation of EV-based biomarkers. MISEV 2023 guidelines recommend the use of orthogonal methods for EV characterization, including morphological analysis, size distribution and particle concentration measurement, protein-based verification using EV-enriched markers from multiple categories, and assessment of non-EV co-isolates to evaluate preparation purity [91]. Fifty-three studies (82.8%) met these minimum reporting requirements. The remaining 11 studies (17.2%) did not report EV-associated markers, which limited interpretation and reproducibility.

Heterogeneity in isolation methods, characterization standards, and analytical platforms complicates cross-study comparisons and may limit reproducibility of reported biomarker associations. Future studies should prioritize standardized experimental protocols, transparent characterization reporting aligned with MISEV guidelines, and prospective validation in independent cohorts to improve rigor and support clinical evaluation.

### 4.4. Geographic and Biological Concentration: Asia and TNBC

Most included studies were conducted in Asia (60.9%, 39/64), followed by Europe (17.2%, 11/64) and North America (12.5%, 8/64). Within Asia, China accounted for the largest proportion of studies (51.6%, 33/64), followed by South Korea (6.2%, 4/64) and Japan (3.1%, 2/64). The remaining studies originated from Brazil (4.7%, 3/64), Turkey (1.6%, 1/64), Russia (1.6%, 1/64), and Egypt (1.6%, 1/64) (Figure 2). This geographic concentration may reflect the availability of large, well-characterized patient cohorts and greater investment in translational and precision oncology research. However, the underrepresentation of Western and ethnically diverse populations warrants attention, because cross-population differences in tumor biology, treatment protocols, and genomic background may limit generalizability. Broader geographic representation and cross-population validation will be important to support broader applicability.

From a biological perspective, TNBC was the most common exclusive research target (15.6%, 10/64), which may reflect the unmet need for predictive biomarkers in this subtype, given its aggressive clinical behavior and limited targeted treatment options [92]. In contrast, HR-positive/luminal (7.8%, 5/64) and HER2-positive (3.1%, 2/64) subtypes were less frequently studied as exclusive targets, indicating opportunities for expanded subtype-specific investigation. The remaining 47 studies (73.4%) enrolled mixed or all-subtype populations, which may improve generalizability but can obscure subtype-specific biomarker performance.

### 4.5. Multi-Omics EV Biomarkers

The findings of this review suggest that integrated multi-analyte EV profiling may improve predictive performance relative to single-analyte approaches. In several studies, simultaneous analysis of multiple EV cargo types, such as miRNA, lncRNA, and surface proteins, was associated with improved discriminatory accuracy. Kim et al. [32] reported that integrating EV surface proteins (MDR1, MRP1, and BCRP) with miRNA cargo yielded higher AUC values for predicting both pCR and PFS than either modality alone. Similarly, combined profiling of EV-associated miR-19a and serum IBSP protein was reported to improve the prediction of bone metastasis [64].

The rationale for multimodal profiling is supported by the complementary nature of EV cargo, which may reflect diverse biological processes, including transcriptional regulation, post-translational signaling, and genomic instability [10]. Each analyte captures only part of tumor biology, whereas integrated approaches may provide a more comprehensive representation.

This complementarity extends beyond EV cargo itself: when considered alongside other liquid biopsy components, EVs occupy a distinct biological niche. While ctDNA provides a genomic snapshot primarily derived from dying cells, and CTCs reflect the cellular phenotype associated with metastatic dissemination, EVs uniquely capture the functional state and active signaling networks of viable tumor cells [93]—offering information that neither ctDNA nor CTCs can fully provide. EVs should therefore be positioned not as a replacement for these established analytes, but as a complementary platform that adds a distinct and orthogonal dimension to tumor characterization [84].

Despite these advantages, head-to-head comparative studies involving EVs, ctDNA, and CTCs within the same patient cohorts remain scarce, and the added clinical value of multi-analyte integration over individual components has yet to be rigorously established. Emerging single-EV multi-omics technologies—such as nano-flow cytometry, proximity barcoding assays, and dielectrophoresis (DEP)-based platforms—capable of simultaneously quantifying surface proteins and internal transcriptomes at the individual vesicle level represent a promising direction for advancing liquid biopsy [94]. These platforms may enable the identification of clinically relevant EV subpopulations that are not detectable using conventional bulk methods.

Nevertheless, the clinical translation of integrated multi-analyte platforms will require overcoming several important barriers. Standardized protocols for the co-isolation and co-analysis of EVs, ctDNA, and CTCs are currently lacking, and robust bioinformatic pipelines capable of handling high-dimensional multi-omics data remain underdeveloped. Prospective validation in large, ethnically diverse cohorts will also be essential before such approaches can be adopted in routine clinical practice.

Taken together, future EV biomarker research in breast cancer should move beyond single-analyte discovery toward development and validation of integrated multimodal panels that encompass the genetic landscape (ctDNA), phenotypic insights (CTCs), and functional signaling (EVs), with an emphasis on standardization and clinical translation.

### 4.6. Artificial Intelligence-Enabled Analytical Technologies for EV Biomarker Applications

Recent advances in artificial intelligence (AI)-enabled analytical technologies represent a promising direction for enhancing the clinical utility of EV-based biomarkers. Deep learning approaches have increasingly been integrated with biosensor platforms and point-of-care testing systems to improve signal interpretation, quantification accuracy, and analytical sensitivity. For example, computational frameworks combining time-lapse imaging with gold nanoparticle amplification chemistry have achieved sub-pg/mL detection limits for protein biomarkers in patient serum [95], while deep learning-based lateral flow assay platforms integrating spatial and temporal feature extraction demonstrated excellent quantitative performance in clinical blind testing [96]. Although these approaches have primarily been evaluated in non-EV biomarker settings, the underlying principles of AI-assisted signal decoding, automated pattern recognition, and multiplex classification are highly applicable to EV-based liquid biopsy platforms, where heterogeneous cargo composition and complex multidimensional datasets present substantial analytical challenges. Integration of AI-enabled analytical systems may facilitate more standardized, sensitive, and scalable implementation of EV-based diagnostics in future clinical practice.

### 4.7. Limitations and Future Directions

This review has several limitations that should be considered when interpreting its findings. First, as a scoping review, formal quality appraisal of individual studies was not performed, and publication bias cannot be excluded. The findings therefore represent a mapping of the published evidence rather than a quantitative synthesis of effect estimates. Second, many included studies were based on relatively small patient cohorts and retrospective designs, which may limit the robustness and generalizability of the reported biomarker associations. Third, substantial heterogeneity in EV isolation methods, biomarker types, analytical platforms, and clinical endpoints precluded formal meta-analytic synthesis and limited direct cross-study comparisons. Fourth, the predominance of prognostic over treatment-response studies in the included literature represents an important limitation in interpreting the full clinical utility of EV biomarkers across different applications. This imbalance likely reflects the current evidence landscape in the field, where prognostic endpoints such as overall survival and disease-free survival are more readily evaluated using retrospective longitudinal cohorts. In contrast, rigorous assessment of treatment response often requires prospective study designs incorporating serial EV sampling and standardized collection protocols, which remain relatively limited. Consequently, the comparative maturity of evidence supporting EV biomarkers for prognostic versus treatment-response applications could not be fully assessed in this review, and conclusions regarding treatment monitoring should therefore be interpreted with caution. Future prospective studies incorporating longitudinal sampling strategies will be essential to address this gap. Finally, the concentration of studies in East Asian cohorts may restrict the external validity of the current evidence base.

Several translational priorities must be addressed before EV biomarkers can be implemented clinically. Large-scale prospective validation studies across independent cohorts that are ethnically and clinically diverse represent the most immediate need. In parallel, improvements in EV isolation technologies that balance purity with clinical scalability, together with the development of standardized analytical frameworks and reference materials, will be important prerequisites for integrating EV-based liquid biopsy into routine clinical practice. Regulatory harmonization and multi-institutional adoption will further support translational progress. Collectively, addressing these challenges will be important for realizing the clinical potential of blood-derived EV biomarkers in precision breast cancer management.

## 5. Conclusions

This scoping review highlights growing evidence supporting blood-derived EV biomarkers as promising analytes for treatment-response assessment and prognostic evaluation in breast cancer. Compared with established liquid-biopsy analytes such as ctDNA and CTCs, EVs may offer biological stability and the ability to capture dynamic molecular signals from the tumor microenvironment. These characteristics suggest that EV-based liquid biopsy may provide complementary information for precision oncology.

Despite these encouraging findings, several challenges remain before EV biomarkers can be routinely implemented in clinical practice. Standardization of EV isolation and characterization protocols, particularly through adherence to MISEV guidelines, is essential to improve reproducibility and comparability across studies. In addition, technological advances, including high-sensitivity nanosensing platforms and microfluidic-based detection systems, may improve analytical efficiency and clinical scalability.

Ultimately, large-scale prospective validation studies across independent patient cohorts will be required to establish the clinical utility of EV-derived biomarkers. The evidence synthesized in this review provides an overview of the current research landscape and may serve as a foundation for future translational studies aimed at integrating EV-based liquid biopsy into personalized breast cancer management.

## Figures and Tables

**Table 1:** Eligibility criteria for the scoping review.

	Inclusion Criteria	Exclusion Criteria
**Population**	Patients diagnosed with breast cancer Any molecular subtype (HR+, HER2+, TNBC) Any disease stage (Early, Locally Advanced, Metastatic)	Non-breast cancer populations Studies with no human clinical subjects
**Outcome**	Association between EV cargo and clinical outcomes Endpoints: Treatment response (pCR, RR) and/or Prognosis (OS, DFS, PFS, RFS)	Focused solely on diagnostic or early detection performance No report on therapeutic or prognostic outcomes
**Publication type**	Original peer-reviewed articles	Non-original studies (e.g., reviews, comments, editorials, notes, case reports, conference abstracts, etc.) Pre-clinical studies (In vitro/In vivo only)
**Language**	English	All other languages

Abbreviations: HR+, hormone receptor–positive; HER2+, human epidermal growth factor receptor 2–positive; TNBC, triple-negative breast cancer; EV, extracellular vesicle; pCR, pathological complete response; RR, response rate; OS, overall survival; DFS, disease-free survival; PFS, progression-free survival; RFS, relapse-free survival.

**Table 4:** Subtype-specific Exosomal Biomarkers and Clinical Significance.

Molecular Subtype	Author (Year)	Key Biomarker (Cargo)	Clinical Significance
TNBC	Todorova (2022) [57]	miR-30b, 141, 34a	Early prediction of pCR using dynamic miRNA changes during NAC.
	Wang (2025) [80]	RTN4 (Protein)	Key driver for metastasis and immune evasion in TNBC.
	Vikramdeo (2023) [59]	EV-mtDNA mutations	Reflects tumor-specific mitochondrial genetic alterations in blood.
	Sueta (2021) [51]	miR-4448, 2392	Prediction of pCR and future recurrence risk in TNBC patients.
HER2+	Liu (2020) [43]	FBXO39 mRNA	High correlation with HER2 expression and Ki-67 index; predicts OS.
	Liu (2023) [41]	lncRNA Linc00969	Transmission of Trastuzumab resistance via exosomal cargos.
	Zhang (2020) [70]	miR-1246, miR-155	High expression in Trastuzumab-resistant cohorts; predictive of efficacy.
	Yang (2025) [68]	Exosomal HER2 protein	Diagnostic value (AUC > 0.85) for HER2+ breast cancer detection.
HR+	Richard (2024) [46]	EV-sphingo scores	Prediction of early resistance to CDK4/6 inhibitors (Palbociclib).
	Del Re (2019) [78]	TK1 & CDK9 mRNA	Monitoring therapeutic response to CDK4/6 inhibitors in metastatic BC.
	Sun (2023) [52]	tRF-16-K8J7K1B	Exosomal transfer of Tamoxifen resistance in HR+ breast cancer.
	Wang (2021) [61]	miR-363-5p	Tumor suppressor role; inhibition of lymph node metastasis.

Abbreviations: HR+, hormone receptor–positive; HER2+, human epidermal growth factor receptor 2–positive; TNBC, triple-negative breast cancer; mRNA, messenger RNA; miRNA, microRNA; lncRNA, long non-coding RNA; tRF, transfer RNA–derived fragments; OS, overall survival.

**Table 5:** Detailed Analysis of Longitudinal Monitoring Studies.

Author (Year)	Biomarker(s)	Sampling Timepoints	Key Longitudinal Finding & Clinical Link
Liu (2020) [43]	FBXO39 mRNA	Baseline vs. Post-surgery	Treatment response is reflected by a significant decrease in mRNA levels; baseline high expression predicts poor prognosis.
Todorova (2022) [57]	miR-141, 34a, 182, 183	Baseline vs. After 1st Cycle	Dynamic changes in miRNA profiles after the first cycle of NAC can predict pCR achievement early.
Cui (2020) [22]	LDHC mRNA	Pre-op, Post-op, Recurrence	Marker levels decrease significantly after surgery and surge again upon recurrence, useful for monitoring relapse.
Sueta (2021) [51]	miR-4448, miR-2392, etc.	Baseline vs. Post-NAC	Pre-treatment profiles predict pCR, while post-treatment changes assess the risk of future recurrence.
Richard (2024) [46]	16 EV-sphingo scores	Baseline vs. 2 months post-Tx	Sphingolipid signatures (Ceramide/SM) within EVs after 2 months of CDK4/6 inhibitors predict early drug resistance.
Tian (2021) [55]	8-EV protein signature	Repeated cycles (Dynamic)	Serial profiling of surface proteins accurately reflects real-time therapeutic response in metastatic patients. 8-EV protein signature marker (EV CA 15-3, CA 125, CEA, HER2, EGFR, PSMA, EpCAM, and VEGF)

Abbreviations: mRNA, messenger RNA; miRNA, microRNA; NAC, Neoadjuvant chemotherapy; op, operation; Tx, treatment, pCR, pathological complete response; EV, extracellular vesicle.

## Data Availability

Data sharing is not applicable to this article because no datasets were generated or analyzed in this study.

## Supplementary Materials

The following supporting information can be downloaded at: https://www.mdpi.com/article/10.3390/ijms27104649/s1.

## Abbreviations

The following abbreviations are used in this manuscript:

CA15-3	Cancer antigen 15-3
CEA	Carcinoembryonic antigen
cfDNA	Cell-free DNA
circRNA	Circular RNA
CTCs	Circulating tumor cells
ctDNA	Circulating tumor DNA
DFS	Disease-free survival
DNA	Deoxyribonucleic acid
EV	Extracellular vesicle
HER2+	Human epidermal growth factor receptor 2-positive
HR+	Hormone receptor-positive
JBI	Joanna Briggs Institute
lncRNA	Long non-coding RNA
mRNA	Messenger RNA
miRNA	MicroRNA
MISEV	Minimal Information for Studies of Extracellular Vesicles
NTA	Nanoparticle tracking analysis
OS	Overall survival
PCC	Population–Concept–Context
PFS	Progression-free survival
pCR	Pathological complete response
PRISMA-ScR	Preferred Reporting Items for Systematic Reviews and Meta-Analyses extension for Scoping Reviews
RFS	Relapse-free survival
RNA	Ribonucleic acid
SEC	Size-exclusion chromatography
TEM	Transmission electron microscopy
TNBC	Triple-negative breast cancer
tRF	Transfer RNA-derived fragments
UC	Ultracentrifugation
WB	Western blotting
