# Supplementary material for: Clinical Applications of Blood-Derived Extracellular Vesicle Biomarkers in Breast Cancer: A Scoping Review
Source: Int J Mol Sci. 2026 May 21;27(10):4649. doi: 10.3390/ijms27104649 (PMC13206953; doi:10.3390/ijms27104649)
Supplement: Supplementary file 1 [file ijms-27-04649-s001.zip › Supplementary Tables.pdf]

Supplementary Table S1. Full Electronic Search Strategies

#	Database	Search Terms	Filters Applied	Date Searched	Results (n)
1	**PubMed**	("breast cancer"[All Fields] OR "breast neoplasms"[All Fields] OR "mammary carcinoma"[All Fields]) OR "Breast Neoplasms"[MeSH Terms]) AND ("exosome"[All Fields] OR "exosomes"[All Fields] OR "extracellular vesicle"[All Fields] OR "EV"[All Fields] OR “exosome cargo”[All Fields] OR “liquid biopsy”[All Fields] OR "extracellular vesicles"[MeSH Terms] OR “exosomes”[MeSH Terms] OR “Liquid Biopsy”[MeSH Terms]) AND ("biomarker"[All Fields] OR “tissue-specific biomarker”[All Fields] OR “circulating biomarker”[All Fields] OR "miRNA"[All Fields] OR "microRNA"[All Fields] OR "exosomal protein"[All Fields] OR "biomarkers"[MeSH Terms] OR "micrornas"[MeSH Terms] OR "proteins"[MeSH Terms]) AND ("diagnosable"[All Fields] OR "diagnosis"[All Fields] OR "prognosis"[All Fields] OR "survival"[All Fields] OR "recurrence"[All Fields] OR "treatment response"[All Fields] OR "therapy resistance"[All Fields] OR "diagnosis"[MeSH Terms] OR "prognosis"[MeSH Terms] OR "therapeutics"[MeSH Terms] OR "recurrence"[MeSH Terms]) AND (“body fluid”[All Fields] OR "blood"[All Fields] OR "serum"[All Fields] OR "plasma"[All Fields] OR “tissue”[All Fields] OR “body fluids”[MeSH Terms]) NOT (review[pt] OR systematic review[pt] OR editorial[pt] OR comment[pt] OR case reports[pt])	English; up to October 2025	2025.11.11	597
2	**Embase**	(breast NEXT cancer:ti,ab,kw OR "breast neoplasm*":ti,ab,kw OR "mammary carcinoma*":ti,ab,kw) AND (exosome*:ti,ab,kw OR "extracellular vesicle*":ti,ab,kw OR EV:ti,ab,kw OR "exosome NEAR/3 cargo":ti,ab,kw OR "liquid biopsy":ti,ab,kw) AND (diagnosis:ti,ab,kw OR prognosis:ti,ab,kw OR survival:ti,ab,kw OR recurrence:ti,ab,kw OR "treatment NEAR/3 response":ti,ab,kw OR "therapy NEAR/3 resistance":ti,ab,kw) NOT (review:pt OR "systematic review":pt OR editorial:pt OR comment:pt OR "case report":pt)	English; up to October 2025	2025.11.11	295
3	**Web of Science**	("breast cancer" OR "breast neoplasm*" OR "mammary carcinoma*")AND (exosome* OR "extracellular vesicle*" OR "liquid biopsy") AND (biomarker* OR "microRNA*" OR miRNA* OR "exosomal protein*") AND (diagnosis OR prognosis OR survival OR recurrence OR "treatment response" OR "therapy resistance")	English; up to October 2025	2025.11.11	963

#	Database	Search Terms	Filters Applied	Date Searched	Results (n)
		**Total retrieved (before deduplication)**			1855
		After deduplication			1529
		After title/abstract screening			189
		After full-text eligibility review			64

Abbreviations: MeSH = Medical Subject Headings; [All Fields] = PubMed all-fields tag; ti,ab,kw = title, abstract, keyword fields (Embase); pt = publication type; NEXT = adjacent word operator; NEAR/3 = within 3 words.

Search date: 11 November 2025. Language restriction: English only. Publication types excluded: reviews, systematic reviews, editorials, comments, and case reports. * indicates truncation.

Supplementary Table S2. List of articles excluded at the stage of full-text assessment

No	Reference	Title	Reason for exclusion
1	C.Chung Park et al. (2024)	All-in-One Fusogenic Nanoreactor for the Rapid Detection of Exosomal MicroRNAs for Breast Cancer Diagnosis	in vitro or experimental study
2	K. L.Wang Zhu et al. (2022)	Analysis of Competitive Endogenous RNA Regulatory Network of Exosomal Breast Cancer Based on exoRBase	in vitro or experimental study
3	Y. C. Z.Su Xie et al. (2022)	Artificial Intelligent Label-Free SERS Profiling of Serum Exosomes for Breast Cancer Diagnosis and Postoperative Assessment	Not related based on subject
4	M.Brown Hu et al. (2023)	Assessing Breast Cancer Molecular Subtypes Using Extracellular Vesicles' mRNA	Not related based on subject
5	J.Kwon An et al. (2025)	Association between breast cancer risk factors and blood microbiome in patients with breast cancer	Not related based on subject
6	K. D.Liu Li et al. (2025)	ATG7-deficient fibroblast promotes breast cancer progression via exosome-mediated downregulation of SCARB1	in vitro or experimental study
7	H.Salimian Sayaf et al. (2025)	Botox-A induced apoptosis and suppressed cell proliferation in fibroblasts pre-treated with breast cancer exosomes	in vitro or experimental study
8	K. M.Marini Bussard et al. (2015)	Breast cancer cell quiescence in bone is orchestrated by osteoblasts transitioned into Tumor-Associated Fibroblasts via exosomal miRs	Not related based on subject
9	C.Zhao Lu et al. (2021)	Breast cancer cell-derived extracellular vesicles transfer miR-182-5p and promote breast carcinogenesis via the CMTM7/EGFR/AKT axis	in vitro or experimental study
10	Y.Zhai Chen et al. (2021)	Breast cancer plasma biopsy by in situ determination of exosomal microRNA-1246 with a molecular beacon	in vitro or experimental study
11	Y.Xu Pan et al. (2022)	A Breast Cancer Prediction Model Based on a Panel from Circulating Exosomal miRNAs	Not related based on subject
12	S. W.Lima Wen et al. (2019)	Breast Cancer-Derived Exosomes Reflect the Cell-of-Origin Phenotype	in vitro or experimental study

13	C.Fang Ni et al. (2020)	Breast cancer-derived exosomes transmit lncRNA SNHG16 to induce CD73+γδ1 Treg cells	in vitro or experimental study
14	M.Regondi Di Modica et al. (2017)	Breast cancer-secreted miR-939 downregulates VE-cadherin and destroys the barrier function of endothelial monolayers	Not related based on subject
15	N.Agarwal Guzman et al. (2015)	Breast Cancer-Specific miR Signature Unique to Extracellular Vesicles Includes microRNA-like" tRNA Fragments"	Not related based on subject
16	A.Philley Kannan et al. (2019)	Cancer Testis Antigen Promotes Triple Negative Breast Cancer Metastasis and is Traceable in the Circulating Extracellular Vesicles	Not related based on subject
17	C. H.Ma Hsu et al. (2022)	Cancer-Associated Exosomal CBFB Facilitates the Aggressive Phenotype, Evasion of Oxidative Stress, and Preferential Predisposition to Bone Prometastatic Factor of Breast Cancer Progression	Not related based on subject
18	Y.Huang Gao et al. (2025)	Cancer-associated fibroblast-secreted exosomal miR-454-3p inhibits lipid metabolism and ferroptosis in breast cancer by targeting ACSL4	Not related based on subject
19	E.Fiore Donnarumma et al. (2017)	Cancer-associated fibroblasts release exosomal microRNAs that dictate an aggressive phenotype in breast cancer	in vitro or experimental study
20	X.Deng Chen et al. (2022)	Cancer-Derived Small Extracellular Vesicles PICKER	in vitro or experimental study
21	Y. L.Hua Liu et al. (2021)	Carcinoma associated fibroblasts small extracellular vesicles with low miR-7641 promotes breast cancer stemness and glycolysis by HIF-1α	Not related based on subject
22	X. Q.Tang Xiao et al. (2024)	Cascade CRISPR/Cas12a and DSN for the electrochemical biosensing of miR-1246 in BC-derived exosomes	in vitro or experimental study
23	Y.Li Gao et al. (2020)	CD63+Cancer-Associated Fibroblasts Confer Tamoxifen Resistance to Breast Cancer Cells through Exosomal miR-22	Not related based on subject
24	G.Daisy Morad et al. (2020)	Cdc42-Dependent Transfer of mir301 from Breast Cancer-Derived Extracellular Vesicles Regulates the Matrix Modulating Ability of Astrocytes at the Blood-Brain Barrier	Not related based on subject
25	P. J.Guo Qiu et al. (2021)	Characterization of Exosome-Related Gene Risk Model to Evaluate the Tumor Immune Microenvironment and Predict Prognosis in Triple-Negative Breast Cancer	Not related based on subject

26	D. X.Zhang Wei et al. (2025)	CircDUSP16 mediates the effect of triple-negative breast cancer in pirarubicin via the miR-1224-3p/TFDP2 axis	Not related based on subject
27	T.Wang Chen et al. (2021)	CircHIF1A regulated by FUS accelerates triple-negative breast cancer progression by modulating NFIB expression and translocation	in vitro or experimental study
28	W.Liu Zhang et al. (2021)	CircRNA circFOXK2 facilitates oncogenesis in breast cancer via IGF2BP3/miR-370 axis	in vitro or experimental study
29	X. R.Liu Ma et al. (2020)	circRNA-associated ceRNA network construction reveals the circRNAs involved in the progression and prognosis of breast cancer	Not related based on subject
30	J. H.Li Ji et al. (2025)	circSTIL mediates pirarubicin inhibiting the malignant phenotype of triple-negative breast cancer and acts as a biomarker in plasma exosomes	Not related based on subject
31	Y.Wang Li et al. (2024)	CircTRIM1 encodes TRIM1-269aa to promote chemoresistance and metastasis of TNBC via enhancing CaM-dependent MARCKS translocation and PI3K/AKT/mTOR activation	Not related based on subject
32	C.Dwyer O'Neill et al. (2019)	Circulating extracellular vesicle (EV)-encapsulated microRNAs as a biomarker of breast cancer	Not related based on subject
33	J.Leal-Orta Ramírez-Ricardo et al. (2020)	Circulating extracellular vesicles from patients with breast cancer enhance migration and invasion via a Src-dependent pathway in MDA-MB-231 breast cancer cells	in vitro or experimental study
34	M. H.Zhou Li et al. (2018)	Circulating microRNAs from the miR-106a-363 cluster on chromosome X as novel diagnostic biomarkers for breast cancer	Not related based on subject
35	E.Luengo Gil García Garre et al. (2018)	Circulating small-sized endothelial microparticles as predictors of clinical outcome after chemotherapy for breast cancer: an exploratory analysis	Not related based on subject
36	Z.Hidalgo Andreu et al. (2024)	Comparative profiling of whole-cell and exosome samples reveals protein signatures that stratify breast cancer subtypes	Not related based on subject
37	K.Quintavalle Pane et al. (2022)	Comparative Proteomic Profiling of Secreted Extracellular Vesicles from Breast Fibroadenoma and Malignant Lesions: A Pilot Study	in vitro or experimental study
38	S. C.Tu Shen et al. (2023)	Comparative Proteomics Analysis of Exosomes Identifies Key Pathways and Protein Markers Related to Breast Cancer Metastasis	in vitro or experimental study

39	D.Polat Ayan et al. (2025)	Comparison Bioinformatic Analysis of Extracellular Vesicles-Related Genes and MicroRNAs in Breast Cancer	Not related based on subject
40	Y. J.Ni Lee et al. (2023)	Comparison Study of Small Extracellular Vesicle Isolation Methods for Profiling Protein Biomarkers in Breast Cancer Liquid Biopsies	Not related based on subject
41	S. S.Cheng Mao et al. (2023)	Comprehensive analysis of the exosomal circRNA-miRNA-mRNA network in breast cancer	Not related based on subject
42	A.Kasyanchyk Khraibah et al. (2025)	Comprehensive Proteomic Profiling of Triple-Negative Breast Cancer-Derived Small Extracellular Vesicles Unveiled PXDN and GGT5 as Novel Protein Markers Implicated in Oncogenic Signaling Networks	in vitro or experimental study
43	Q.Wang Wu et al. (2019)	Construction of an Autonomous Nonlinear Hybridization Chain Reaction for Extracellular Vesicles-Associated MicroRNAs Discrimination	in vitro or experimental study
44	L. M.Zhang Yang et al. (2025)	CRISPR-Based Homogeneous Electrochemical Strategy for Near-Zero Background Detection of Breast Cancer Extracellular Vesicles via Fluidity-Enhanced Magnetic Capture Nanoprobe	in vitro or experimental study
45	D. D.Li Liu et al. (2021)	Cross-platform genomic identification and clinical validation of breast cancer diagnostic biomarkers	Not related based on subject
46	H. H.Kim Jung et al. (2020)	Cytokine profiling in serum-derived exosomes isolated by different methods	in vitro or experimental study
47	D.Wijesinghe Pokharel et al. (2016)	Deciphering Cell-to-Cell Communication in Acquisition of Cancer Traits: Extracellular Membrane Vesicles Are Regulators of Tissue Biomechanics	in vitro or experimental study
48	G. B.Wang Zhong et al. (2020)	Determination of Serum Exosomal H19 as a Noninvasive Biomarker for Breast Cancer Diagnosis	Not related based on subject
49	R.Rezaie Asgari et al. (2022)	Differential Expression of Serum Exosomal miRNAs in Breast Cancer Patients and Healthy Controls	Not related based on subject
50	C.Tanaka Jinno et al. (2024)	Discovery of liquid biopsy-based novel protein biomarkers for early detection of breast cancer metastasis	Not related based on subject
51	E.Rossi Bandini et al. (2021)	Early Detection and Investigation of Extracellular Vesicles Biomarkers in Breast Cancer	Not related based on subject
52	M.Ji Wang et al. (2018)	Effect of exosome biomarkers for diagnosis and prognosis of breast cancer patients	Review study, editorial, letter, or meeting abstract

53	S. R.Liu Wang et al. (2025)	Efficient metabolic fingerprinting profiling of extracellular vesicles for precise cancer diagnosis and treatment monitoring	Not related based on subject
54	S. L.Martín Moura et al. (2020)	Electrochemical immunosensing of nanovesicles as biomarkers for breast cancer	in vitro or experimental study
55	K. P.Khan O'Brien et al. (2018)	Employing mesenchymal stem cells to support tumor-targeted delivery of extracellular vesicle (EV)-encapsulated microRNA-379	Not related based on subject
56	A.Khodadadi Asadirad et al. (2022)	Evaluation of miRNA-21-5p and miRNA-10b-5p levels in serum-derived exosomes of breast cancer patients in different grades	Not related based on subject
57	S.Chaudhary Maji et al. (2017)	Exosomal Annexin II Promotes Angiogenesis and Breast Cancer Metastasis	Not related based on subject
58	S. J.Lee Lee et al. (2021)	Exosomal Del-1 as a Potent Diagnostic Marker for Breast Cancer: Prospective Cohort Study	Not related based on subject
59	J. Y.Shih Kan et al. (2024)	Exosomal microRNA-92b Is a Diagnostic Biomarker in Breast Cancer and Targets Survival-Related MTSS1L to Promote Tumorigenesis	Not related based on subject
60	C. C.Osmanlioglu Serdar et al. (2023)	Exosomal prognostic biomarkers predict metastatic progression and survival in breast cancer patients	Review study, editorial, letter, or meeting abstract
61	C. C.Su Hsu et al. (2024)	Exosomal Thomsen-Friedenreich Glycoantigen: A New Liquid Biopsy Biomarker for Lung and Breast Cancer Diagnoses	Not related based on subject
62	C. P.Zhan Lu et al. (2023)	Exosome-derived ANXA9 functions as an oncogene in breast cancer	Not related based on subject
63	J. J.Xu Ding et al. (2018)	Exosome-mediated miR-222 transferring: An insight into NF-κ-mediated breast cancer metastasis	Not related based on subject
64	B.Mao Wang et al. (2024)	Exosome-mediated transfer of lncRNA RP3-340B19.3 promotes the progression of breast cancer by sponging miR-4510/MORC4 axis	Not related based on subject
65	R.Pochampally Singh et al. (2014)	Exosome-mediated transfer of miR-10b promotes cell invasion in breast cancer	Not related based on subject

66	D. B.Ghate Alagundagi et al. (2023)	Exploring breast cancer exosomes for novel biomarkers of potential diagnostic and prognostic importance	in vitro or experimental study
67	H.Limon-Miro Mendivil-Alvarado et al. (2023)	Extracellular Vesicles and Their Zeta Potential as Future Markers Associated with Nutrition and Molecular Biomarkers in Breast Cancer	Not related based on subject
68	P.Sadovska Zayakin et al. (2023)	Extracellular Vesicles-A Source of RNA Biomarkers for the Detection of Breast Cancer in Liquid Biopsies	Not related based on subject
69	J.Guan Zhang et al. (2023)	Highly Effective Detection of Exosomal miRNAs in Plasma Using Liposome-Mediated Transfection CRISPR/Cas13a	in vitro or experimental study
70	Q. A.Pan Guo et al. (2023)	Identification of an exosome-related signature associated with prognosis and immune infiltration in breast cancer	in vitro or experimental study
71	S. R.Yeung Douglas et al. (2021)	Identification of CD105+ Extracellular Vesicles as a Candidate Biomarker for Metastatic Breast Cancer	Not related based on subject
72	S. J.Wang Yang et al. (2020)	Identification of circRNA-miRNA networks for exploring an underlying prognosis strategy for breast cancer	Not related based on subject
73	Y.Wang Xin et al. (2020)	Identification of exosomal miR-455-5p and miR-1255a as therapeutic targets for breast cancer	in vitro or experimental study
74	Y.Wang Cao et al. (2020)	Identification of programmed death ligand-1 positive exosomes in breast cancer based on DNA amplification-responsive metal-organic frameworks	in vitro or experimental study
75	Y.Pan Zhang et al. (2023)	Identifying tumor cell-released extracellular vesicles as biomarkers for breast cancer diagnosis by a three-dimensional hydrogel-based electrochemical immunosensor	in vitro or experimental study
76	C.Stückrath Eichelser et al. (2014)	Increased serum levels of circulating exosomal microRNA-373 in receptor-negative breast cancer patients	Not related based on subject
77	J.Sirati-Sabet Razaviyan et al. (2024)	Inhibition of MiR-155 Using Exosomal Delivery of Antagomir Can Up-Regulate PTEN in Triple Negative Breast Cancer	Not related based on subject

78	Y.Chen Qiu et al. (2025)	Insulin Resistance Increases TNBC Aggressiveness and Brain Metastasis via Adipocyte-Derived Exosomes	Not related based on subject
79	I.Perego Bertolini et al. (2023)	Intercellular HIF1? reprograms mammary progenitors and myeloid immune evasion to drive high-risk breast lesions	Not related based on subject
80	Z. Q.Zhang Meng et al. (2024)	LncRNA HAGLROS promotes breast cancer evolution through miR-135b-3p/COL10A1 axis and exosome-mediated macrophage M2 polarization	Not related based on subject
81	J. L.Li Mo et al. (2024)	A machine learning model revealed that exosome small RNAs may participate in the development of breast cancer through the chemokine signaling pathway	Not related based on subject
82	L.Guerrero Camacho et al. (2013)	MicroRNA and Protein Profiling of Brain Metastasis Competent Cell-Derived Exosomes	in vitro or experimental study
83	S. L.Chen Zhong et al. (2016)	MicroRNA expression profiles of drug-resistance breast cancer cells and their exosomes	Not related based on subject
84	I.Godinho-Pereira Figueira et al. (2021)	MicroRNAs and Extracellular Vesicles as Distinctive Biomarkers of Precocious and Advanced Stages of Breast Cancer Brain Metastases Development	in vitro or experimental study
85	S.Olioso Catelan et al. (2022)	miRNAs in Serum Exosomes for Differential Diagnosis of Brain Metastases	Not related based on subject
86	F.Ma Long et al. (2023)	A novel exosome-derived prognostic signature and risk stratification for breast cancer based on multi-omics and systematic biological heterogeneity	Not related based on subject
87	M. W.Xia Zhang et al. (2023)	One-step multiplex analysis of breast cancer exosomes using an electrochemical strategy assisted by gold nanoparticles	in vitro or experimental study
88	Z. Y.Yin Gu et al. (2022)	Optimization of a method for the clinical detection of serum exosomal miR-940 as a potential biomarker of breast cancer	in vitro or experimental study
89	I. H.Xue Chen et al. (2017)	Phosphoproteins in extracellular vesicles as candidate markers for breast cancer	Not related based on subject
90	X.Qian Wang et al. (2019)	Phosphorylated Rasal2 facilitates breast cancer progression	in vitro or experimental study

91	B. N.Trigoso Hannafon et al. (2016)	Plasma exosome microRNAs are indicative of breast cancer	Not related based on subject
92	T.Naryzhny Shtam et al. (2019)	Plasma exosomes stimulate breast cancer metastasis through surface interactions and activation of FAK signaling	in vitro or experimental study
93	G. X.Li Cai et al. (2021)	A plasma-derived extracellular vesicle mRNA classifier for the detection of breast cancer	Not related based on subject
94	L.Cai Lin et al. (2021)	Plasma-Derived Extracellular Vesicles Circular RNAs Serve as Biomarkers for Breast Cancer Diagnosis	Not related based on subject
95	A.Bhardwaj Gupta et al. (2025)	Potential applications of gene expression profiles obtained from circulating extracellular vesicles in breast cancer	Not related based on subject
96	Z. J.Wang Zhang et al. (2022)	Potential of blood exosomal ENAH, SEPT9, EGF, MMP-9 and CXCL8 for the early screening of breast cancer	Not related based on subject
97	J.Goh Howard et al. (2022)	The potential role of cofilin-1 in promoting triple negative breast cancer (TNBC) metastasis via the extracellular vesicles (EVs)	Not related based on subject
98	J.Shen Zhao et al. (2024)	Prognostic value and microenvironmental crosstalk of exosome-related signatures in human epidermal growth factor receptor 2 positive breast cancer	Not related based on subject
99	G.Albanese Palazzolo et al. (2012)	Proteomic analysis of exosome-like vesicles derived from breast cancer cells	in vitro or experimental study
100	S.Li Li et al. (2021)	Proteomic Landscape of Exosomes Reveals the Functional Contributions of CD151 in Triple-Negative Breast Cancer	Not related based on subject
101	S.Synadaki Rontogianni et al. (2019)	Proteomic profiling of extracellular vesicles allows for human breast cancer subtyping	Not related based on subject
102	G. C.Hampton Clark et al. (2022)	Radiation induces ESCRT pathway dependent CD44v3+ extracellular vesicle production stimulating pro-tumor fibroblast activity in breast cancer	Not related based on subject

103	C.Shao Pan et al. (2023)	Radiation prevents tumor progression by inhibiting the miR-93-5p/EphA4/NF-κB pathway in triple-negative breast cancer	Not related based on subject
104	X. M.Cao Yu et al. (2026)	Relay race-like" labeling-assisted extracellular vesicles profiling in breast cancer diagnosis"	Not related based on subject
105	J.Chi Wu et al. (2023)	RNA in Extracellular vesicles used for the prediction of distant metastasis of breast cancer and the function of TGM2 in promoting breast cancer development	Review study, editorial, letter, or meeting abstract
106	K.Haas-Neill Platko et al. (2019)	The role of circulating extracellular vesicles in breast cancer classification and molecular subtyping	Not related based on subject
107	P.Du Qiao et al. (2024)	Serum exosomal miR-200c is a potential diagnostic biomarker for breast cancer	Not related based on subject
108	S. H.Espinoza-Sánchez Ahmed et al. (2021)	Small extracellular vesicle-encapsulated miR-181b-5p, miR-222-3p and let-7a-5p: Next generation plasma biopsy-based diagnostic biomarkers for inflammatory breast cancer	Not related based on subject
109	N.Zhang Su et al. (2025)	Specific isolation and quantification of PD-L1 positive tumor derived exosomes for accurate breast cancer discrimination via aptamer-functionalized magnetic composites and SERS immunoassay	in vitro or experimental study
110	E.Miki Iwabuchi et al. (2025)	Tetraspanins CD63 and CD81 as potential prognostic biomarkers in breast cancer	Not related based on subject
111	M.Sagaradze Konoshenko et al. (2020)	Total Blood Exosomes in Breast Cancer: Potential Role in Crucial Steps of Tumorigenesis	Not related based on subject
112	Y.Kim Kim et al. (2024)	Tumor-derived EV miRNA signatures surpass total EV miRNA in supplementing mammography for precision breast cancer diagnosis	Not related based on subject
113	S. J.Wang Yang et al. (2021)	Tumor-derived exosomal circPSMA1 facilitates the tumorigenesis, metastasis, and migration in triple-negative breast cancer (TNBC) through miR-637/Akt1/β-catenin (cyclin D1) axis	Not related based on subject
114	D.Chen Xu et al. (2023)	Tumor-derived small extracellular vesicles promote breast cancer progression by upregulating PD-L1 expression in macrophages	Not related based on subject
115	K. A.Wang Ning et al. (2017)	UCH-L1-containing exosomes mediate chemotherapeutic resistance transfer in breast cancer	Not related based on subject

116	S. S.Zhang Cheng et al. (2024)	Ultrasensitive determination of surface proteins on tumor-derived small extracellular vesicles for breast cancer identification based on lanthanide-activated signal amplification strategy	in vitro or experimental study
117	K.Yamamoto Otsuka et al. (2020)	Uncovering temperature-dependent extracellular vesicle secretion in breast cancer	in vitro or experimental study
118	Y. C.Yan Liu et al. (2020)	Aberrant Expression of Cancer-Testis Antigen FBXO39 in Breast Cancer and its Clinical Significance	Not related based on subject
119	I.Gelsomino Barone et al. (2023)	Analysis of circulating extracellular vesicle derived microRNAs in breast cancer patients with obesity: a potential role for Let-7a	Not related based on subject
120	G.Cohen Bar-Sela et al. (2020)	Circulating blood extracellular vesicles as a tool to assess endothelial injury and chemotherapy toxicity in adjuvant cancer patients	Not related based on subject
121	S.Bennit Khan et al. (2014)	Early diagnostic value of survivin and its alternative splice variants in breast cancer	Not related based on subject
122	A.Zeune Nanou et al. (2020)	HER2 expression on tumor-derived extracellular vesicles and circulating tumor cells in metastatic breast cancer	Not related based on subject
123	H.Shu Wang et al. (2022)	Novel lncRNAs with diagnostic or prognostic value screened out from breast cancer via bioinformatics analyses	Not related based on subject
124	C.Hu Yan et al. (2019)	Plasma extracellular vesicle-packaged microRNAs as candidate diagnostic biomarkers for early-stage breast cancer	Not related based on subject
125	H.Hu Hu at al. (2021)	Assessment of circulating HISLA as a potential biomarker for breast cancer diagnosis and prognosis	Not related based on subject

Supplementary Table S3. Technical Characteristics and MISEV Compliance of Included Studies

No.	Author (Year)	Sample Source	Isolation Method	Characterization	Verified EV Markers
1	Curtaz (2022)[23]	Serum	Kit-based	NTA, TEM, WB	CD9, CD63
2	Liu (2020)[43]	Serum	UC + Kit	NTA, TEM, WB	CD9, CD63, TSG101
3	Alvarez (2022)[16]	Plasma	SEC/PPLC	NTA, WB	CD9,CD63, CD81
4	Yuan (2021)[69]	Serum	Kit-based	NTA, TEM, WB	Alix, HSP70, TSG101
5	Li (2024)[38]	Plasma	UC	NTA, TEM, WB	CD63, TSG101
6	Shen (2021)[48]	Plasma	UC	NTA, TEM, WB	CD9, CD63
7	Fan (2025)[25]	Serum	Kit-based	NTA, WB	CD63, CD81,CD9
8	Todorova (2022)[57]	Plasma	Kit-based	N/A	N/A
9	Wang (2021)[61]	Plasma	UC	NTA, TEM, WB	TSG101, CD63
10	Tkach (2022)[56]	Plasma	SEC	NTA, WB	CD63, CD9, MHCI, Syntenin
11	Baldasici (2022)[17]	Plasma	Kit-based	N/A	N/A
12	Desai (2022)[24]	Serum	Kit-based	NTA, TEM, WB	CD9, CD81, TSG101
13	Sadovska (2022)[47]	Plasma	SEC	NTA, TEM, WB	TSG101, CD9
14	Li (2021)[36]	Serum	Kit-based	NTA, TEM, WB	CD63, TSG101
15	Fontana (2025)[26]	Plasma	Kit-based	N/A	N/A
16	Cui (2020)[22]	Serum	Kit-based	TEM, WB	CD9, CD63
17	Ni (2018)[76]	Plasma	Kit-based	WB	CD63
18	Sueta (2017)[73]	Serum	Kit-based	NTA, WB	CD63
19	Wu (2020)[63]	Plasma	Kit-based	NTA	N/A

20	Tamarindo (2025)[54]	Plasma	SEC	NTA, TEM, WB	CD81, ALIX, Syntenin
21	Kim (2024)[32]	Plasma	UC	NTA, TEM,Confocal microscopy	CD63, MDR1
22	Jung (2021)[31]	Serum	UC	NTA, TEM,WB	CD63, CD9, CD81
23	König (2017)[72]	Plasma	Kit-based	NTA, WB, SDS-Page	CD9, CD63, CD81, TSG101
24	Causin (2024)[20]	Plasma	UC	NTA, WB	CALIX, Flotillin-1, CD63
25	Li (2021)[37]	Serum	Kit-based	NTA, TEM, WB	CD63, CD81,CD9
26	Zhuang (2024)[71]	Serum	UC	NTA, TEM, WB	CD9, TSG101
27	Liu (2023)[41]	Serum/Plasma	UC	NTA, TEM, WB	TSG101, CD81, CD9, CD63
28	Wu (2021)[64]	Serum	UC	NTA, WB	CD63, TSG101
29	Li (2024)[39]	Plasma	Kit-based	NTA, TEM, WB	TSG101, HSP70,CD63
30	Zhang (2020)[70]	Plasma	Kit-based	NTA, TEM, WB	CD63, CD81
31	Sueta (2021)[51]	Serum	Kit-based	NTA, WB	CD63
32	Sun (2023)[52]	Serum	UC	NTA, BCA, WB	ALIX, TSG101, CD9, CD63
33	Kim (2024)[33]	Plasma	Immuno-aff	NTA, SEM, Confocal microscopy	CD63
34	Bao (2021)[18]	Serum/Plasma	UC	NTA, TEM, WB	N/A
35	Shi (2022)[49]	Serum	Kit-based	N/A	N/A
36	Li (2020)[35]	Serum	Kit-based	TEM, WB	CD63, CD81
37	Wang (2017)[74]	Plasma	Kit-based	FCM, WB	CD63, Flotillin-1
38	Niu (2025)[45]	Plasma	Kit-based	TEM, WB	CD63, CD9

39	Carvalho (2022)[19]	Serum	Kit-based	NTA	N/A
40	Richard (2024)[46]	Plasma	SEC	Single particle tracking, TEM, WB	Syntenin, CD9
41	Jiang (2024)[29]	Plasma	SEC	NTA, TEM, Dot blots	CD63, CD9, CD81,TSG101
42	Tang (2019)[79]	Serum	Kit-based	TEM, WB	CD63, Hsp70
43	Del Re (2019)[78]	Plasma	Kit-based	NTA, WB	TSG101, CD9, Flotillin-1
44	Yang (2024)[67]	Plasma	Kit-based	NTA, TEM, WB	CD9, CD63, CD81, TSG101
45	Su (2021)[50]	Plasma	Kit-based	Flow NanoAnalyzer, TEM, WB	CD63, TSG101
46	Yang (2025)[68]	Plasma	Kit-based	NTA, TEM, WB	TSG101, CD9, CD63
47	Yang (2017)[75]	Serum	UC	TEM, WB	CD63, TSG101, Alix
48	Vikramdeo (2023)[59]	Plasma	Kit-based	NTA, TEM, WB	CD9, CD63, CD81, TSG101
49	Eskiler (2023)[27]	Serum	Kit-based	Flow Cytometry	CD63, CD81
50	Hoffmann (2023)[28]	Plasma	Kit-based	N/A	N/A
51	Tian (2021)[55]	Plasma	UC	NTA, ELISA	CD63, CD41
52	Vinik (2020)[60]	Plasma	SEC	TEM, WB	TSG101, ALIX, HSP70
53	Tutanov (2020)[58]	Plasma	UC	NTA, TEM, Flow cytometry	N/A
54	Xu (2024)[66]	Serum	UC	TEM, NTA, WB	CD9, CD63, TSG101, ALIX
55	Jung (2023)[30]	Serum	Kit-based	NTA, WB	CD9, CD63, CD81, TSG101, Alix

56	Talat (2025)[53]	Plasma	UC	DLS, TEM, dot blot, WB,ELISA	CD9
57	Lan (2021)[34]	Serum	Kit-based	N/A	N/A
58	Chaudhary (2020)[21]	Serum	Kit-based	Particle size analyzer, WB	AnxA2, TSG101, flotillin-1, calnexin, GM130, EpCAM, CD9
59	Stevic (2018)[77]	Plasma	Kit-based	Confocal microscopy, WB, ELISA	CD9, CD81, TSG101
60	Wang (2025)[80]	Plasma	UC	NanoFCM	CD63, CD81, CD9
61	Li (2024)[40]	Plasma	SEC	NTA, TEM, WB	CD81, HSP90
62	Xu (2024)[65]	Serum	Capture	NTA, TEM, WB	CD63, EpCAM
63	Na-er (2021)[44]	Serum	Kit-based	N/A	N/A
64	Liu (2022)[42]	Plasma	Kit-based	TEM, WB	CD63, CD81, TSG101

Abbreviations: NTA, Nanoparticle Tracking Analysis; TEM, Transmission Electron Microscopy; WB, Western Blot; UC, Ultracentrifugation; SEC, Size Exclusion Chromatography; PPLC, Polymer-based Precipitation and Low-speed Centrifugation; Immuno-aff , Immunoaffinity-based isolation; N/A, Not Applicable
